# L1CAM Expression in Microcystic, Elongated, and Fragmented (MELF) Glands Predicts Lymph Node Involvement in Endometrial Carcinoma

**DOI:** 10.3390/cancers14153635

**Published:** 2022-07-26

**Authors:** Damiano Arciuolo, Antonio Travaglino, Angela Santoro, Giulia Scaglione, Nicoletta D’Alessandris, Michele Valente, Frediano Inzani, Rossella Accarino, Alessia Piermattei, Roberta Benvenuto, Antonio Raffone, Camilla Nero, Silvia Pelligra, Francesco Fanfani, Massimo Mascolo, Gian Franco Zannoni

**Affiliations:** 1 Pathology Unit, Department of Woman and Child’s Health and Public Health Sciences, Fondazione Policlinico Universitario Agostino Gemelli IRCCS, 00168 Rome, Italy; damiano.arciuolo@policlinicogemelli.it (D.A.); antonio.travaglino@guest.policlinicogemelli.it (A.T.); angela.santoro@policlinicogemelli.it (A.S.); giulia.scaglione@policlinicogemelli.it (G.S.); nicoletta.dalessandris@policlinicogemelli.it (N.D.); michele.valente@guest.policlinicogemelli.it (M.V.); frediano.inzani@policlinicogemelli.it (F.I.); alessia.piermattei@policlinicogemelli.it (A.P.); roberta.benvenuto@policlinicogemelli.it (R.B.); gianfranco.zannoni@unicatt.it (G.F.Z.); 2 Department of Life Health and Public Health, Catholic University of the Sacred Hearth, 00168 Rome, Italy; camilla.nero@policlinicogemelli.it; 3 Pathology Unit, Department of Advanced Biomedical Sciences, Federico II University of Naples, 80131 Naples, Italy; rossella.accarino@unina.it; 4 Division of Gynecology and Human Reproduction Physiopathology, Department of Medical and Surgical Sciences (DIMEC), S. Orsola-Malpighi Hospital, University of Bologna, 40138 Bologna, Italy; antonio.raffone2@unibo.it; 5 Gynecologic Oncology Unit, Department of Woman and Child’s Health and Public Health Sciences, Fondazione Policlinico Universitario Agostino Gemelli IRCCS, 00168 Rome, Italy; silvia.pelligra01@icatt.it (S.P.); francesco.fanfani@unicatt.it (F.F.)

**Keywords:** endometrial carcinoma, L1CAM, microcystic, elongated and fragmented, MELF, TCGA, lymph node, prognosis

## Abstract

**Simple Summary:**

L1CAM overexpression (≥10%) and the microcystic, elongated, and fragmented (MELF) pattern of invasion have previously been assessed as prognostic factors in endometrial carcinoma. We aimed to assess the relationship between L1CAM expression, MELF glands, and lymph node involvement in endometrial carcinoma, as all these factors are related to epithelial-to-mesenchymal transition. We evaluated L1CAM expression in 58 cases of uterine-confined, low-grade endometrioid carcinomas. We found that most cases (65.5%) expressed L1CAM in a limited manner to MELF glands. Cases with L1CAM expression in ≥10% of the MELF component showed a significantly higher tendency to lymph node spread (*p* < 0.001), even when adjusted for lymphovascular space invasion, depth of myometrial invasion and p53/mismatch repair status. On this account, L1CAM expression in the MELF component may stratify the prognosis and management in patients with uterine-confined, low-grade carcinomas.

**Abstract:**

In endometrial carcinoma, both L1CAM overexpression and microcystic, elongated and fragmented (MELF) patterns of invasion have been related to epithelial-to-mesenchymal transition and metastatic spread. We aimed to assess the association between L1CAM expression, the MELF pattern, and lymph node status in endometrial carcinoma. Consecutive cases of endometrial carcinoma with MELF pattern were immunohistochemically assessed for L1CAM. Inclusion criteria were endometrioid-type, low-grade, stage T1, and known lymph node status. Uni- and multivariate logistic regression were used to assess the association of L1CAM expression with lymph node status. Fifty-eight cases were included. Most cases showed deep myometrial invasion (*n* = 42, 72.4%) and substantial lymphovascular space invasion (*n* = 34, 58.6%). All cases were p53-wild-type; 17 (29.3%) were mismatch repair-deficient. Twenty cases (34.5%) had positive nodes. No cases showed L1CAM positivity in ≥10% of the whole tumor. MELF glands expressed L1CAM at least focally in 38 cases (65.5%). L1CAM positivity in ≥10% of the MELF component was found in 24 cases (41.4%) and was the only significant predictor of lymph node involvement in both univariate (*p* < 0.001) and multivariate analysis (*p* < 0.001). In conclusion, L1CAM might be involved in the development of the MELF pattern. In uterine-confined, low-grade endometrioid carcinomas, L1CAM overexpression in MELF glands may predict lymph node involvement.

## 1. Introduction

Endometrial carcinoma is the most common gynecological malignancy in developed countries and the second most common worldwide after cervical carcinoma [1]. Endometrial carcinoma mostly occurs in women above the age of 50 [2], with early stage at presentation in more than 90% of cases [3]. In the last several decades, there have been alarming changes in the epidemiology of endometrial carcinoma. In fact, both incidence and mortality of endometrial carcinoma have sensibly increased. Although the increase in incidence might be attributed to the increased frequency of risk factor, the increase in mortality is at least in part attributable to issues in the prognostic stratification and patient management [1,4,5]. According to Bokhman [6], endometrial carcinoma could be divided in two main groups: type 1 endometrial carcinomas typically are progesterone-sensitive, low-grade tumors with and a favorable prognosis; type 2 endometrial carcinomas are progesterone-insensitive, high-grade tumors with an unfavorable prognosis. This dichotomous classification by Bokhman is limited by the molecular heterogeneity present in these tumors.

In 2013, The Cancer Genome Atlas (TCGA) Research Network [7] found four molecular subgroups of endometrial carcinoma: *POLE* mutated/ultramutated, microsatellite unstable/hypermutated, copy number high, and copy number low. This classification allows the stratifying prognosis in endometrial carcinoma [8]. The TCGA findings have highlighted that the traditional risk stratification, based on clinicopathological factors, leads to under- or overtreatment of a considerable proportion of patients with endometrial carcinoma [5,8,9]. The inclusion of patients with different molecular features in clinical trials might also have led to poorly reliable results, based on heterogeneous populations [5]. In recent years, the TCGA classifier has improved the prognostic stratification of patients with endometrial carcinoma [5,8,10]. Such a classifier is progressively being integrated in the common clinical practice and is now recognized by the ESGO-ESTRO-ESP guidelines; traditional pathologic features, such as histotype, grade, myometrial invasion, and lymphovascular space invasion (LVSI), are still considered in the risk-stratification algorithm [8,11]. This process has been facilitated by the use of immunohistochemical surrogates of molecular prognostic markers [8]. Indeed, p53 immunohistochemical assessment is currently used in the place of somatic copy number molecular analysis, and MMR protein immunohistochemical assessment is used in place of microsatellite instability molecular testing [5,8]. Two large study groups, i.e., the Vancouver group from Canada [12,13] and the Leiden group from Netherlands [14,15], have been leaders in this field. There is still no immunohistochemical surrogate for *POLE* mutation, which requires molecular analysis; molecular testing of *POLE* gene is commonly based in the search for hotspot pathogenic mutations [8,11,12,15]. These immunohistochemical and molecular tests allow one to identify four molecular prognostic groups which are surrogates of the TCGA molecular groups: MMR deficient (surrogate of the microsatellite unstable/hypermutated group), p53 abnormal group (surrogate of the copy number high group), no specific molecular profile (NSMP, surrogate of the copy number low group), and *POLE* mutated group (surrogate of the ultramutated group) [8,11,12]. Interestingly, the *POLE* mutated group show good prognosis regardless of clinicopathological features, which not uncommonly are worrisome; for instance, about half of *POLE*-mutated endometrial carcinomas are high-grade [5,8,9]. On the other hand, p53 abnormal expression is associated with poor prognosis, independent of other relevant clinicopathological features [5,8]. MMR-deficient and NSMP groups are considered at intermediate prognosis [5,8,10,11]. 

In the 2020 ESGO-ESTRO-ESP guidelines, all *POLE*-mutated endometrial carcinomas up to FIGO stage II are included in the low-risk prognostic group, regardless of other clinicopathological features, such as tumor grade, histotype, LVSI, and depth of myometrial invasion. On the other hand, all p53 abnormal carcinomas are included in the high-risk prognostic group, except for cases with no myometrial invasion, which are considered at intermediate risk instead; in this way, p53 abnormal endometrioid carcinomas are considered prognostically analogous to serous carcinomas. For the NSMP and MMR-deficient groups, the risk group is based on traditional clinicopathological factors. In particular, cases at FIGO stage IA, endometrioid histotype, and low grade, with no or focal LVSI, are included in the low-risk group; this means that these tumors do not need adjuvant treatment. FIGO IA high-grade endometrioid carcinomas, as well as FIGO IB low-grade endometrioid carcinoma, fall into the intermediate risk group in the case of no or focal LVSI. Endometrioid carcinomas with substantial LVSI (i.e., two or more LVSI foci) fall into the high-intermediate risk group, which also includes endometrioid carcinomas at FIGO stage II [8,11]. Such revised risk stratification, which incorporates the TCGA molecular prognostic groups, is expected to improve oncological outcomes in patients with endometrial carcinomas.

However, there is still a paucity of prospective data from randomized controlled trials in this field. [16] Furthermore, there are several additional prognostic factors that may further refine the prognostic stratification of endometrial carcinomas. These factors might help explain the different clinical course of patients who fall in the same risk group, especially for the NSMP group, which is the most heterogeneous and the least prognostically and biologically defined group [8]. These additional prognostic markers include histomorphological factors, such as tumor budding [17] and microcystic, elongated and fragmented (MELF) pattern of invasion [18]; immunohistochemical factors, such as transmembrane L1 cell adhesion molecule (L1CAM) expression [14] and SWI/SNF complex proteins expression [19]; and molecular factors, such *CTNNB1* exon 3 mutations [14]. 

Among these factors, L1CAM has emerged as one of most promising [8]. L1CAM is a neural adhesion molecule with several functions: in normal tissue, it plays a key role in the development of the nervous system, whereas in cancer it is critical in the process of epithelial to mesenchymal transition [20]. L1CAM induces epithelial to mesenchymal transition through the inhibition of E-Cadherin [21], and its expression increases cell motility and the ability to metastasize. L1CAM overexpression has been associated with poor outcome in endometrial carcinoma, as well as in several other tumors [22,23,24]. Zeimet et al. [25] studied the prognosis related to L1CAM in a large cohort of patients with endometrial carcinoma. They found that L1CAM expression was independently associated with the risk of recurrence and poor prognosis. They found that only the tumors with more than 10% of cells stained for L1CAM had a worse outcome. Subsequently, the trans-PORTEC study from the Leiden group assessed the prognostic value of L1CAM combined with the TCGA groups. The authors found that endometrioid carcinomas with high-intermediate risk clinicopathological features behaved aggressively in the case of L1CAM positivity in more than 10% of tumor cells [14].

The MELF pattern of invasion was first described by Lee, Vacek, and Belinson [26] as an “endothelial-like” pattern of invasion. More recently, Murray et al. coined the term “MELF”. MELF glands are located in the front of invasion of the tumor, and show microcystic, elongated, angulated, and fragmented features and are lined by flat cells or cells with abundant eosinophilic cytoplasm. The glands often contain neutrophils and are surrounded by a desmoplastic or myxoid stroma rich in inflammatory cells [27]. This MELF pattern has been associated with lymph node metastasis [28,29], deep of myometrial invasion, and LVSI [29]. Evidence of epithelial to mesenchymal transition has been found in MELF glands, which is consistent with its tendency to infiltration and nodal spread [30]. Some authors also suggested that the MELF pattern may have a prognostic value independent from the TCGA signatures [18]. However, results regarding the prognostic value of the MELF pattern of invasion in endometrial carcinoma have been inconsistent among published studies [18,28,29,31].

As both L1CAM expression and MELF pattern are related to epithelial to mesenchymal transition and, likely, to metastatic spread, we aimed to assess (i) the relationship between these two factors in endometrial carcinoma and (ii) their association with lymph node metastasis.

## 2. Materials and Methods

Patients were selected from a larger study which assessed L1CAM in a consecutive series of endometrial carcinomas; all patients underwent hysterectomy at the Gynecologic Oncology Unit, Fondazione Policlinico Universitario A. Gemelli, Rome, Italy, with a diagnosis of presumed uterine-confined endometrial carcinoma (IRB approval no. ID3994). Among these patients, we selected all consecutive cases (from January 2018 to October 2021) of low-grade (FIGO G1-G2) endometrioid carcinoma with a MELF pattern of invasion and who underwent pelvic lymphadenectomy or sentinel lymph node biopsy (*n* = 58). The study outcomes were (i) the expression of L1CAM in MELF and (ii) its association with lymph node status.

Histological procedures were performed at the Pathology Unit, Fondazione Policlinico Universitario A. Gemelli, Rome, Italy, as previously described [32]. In brief, hysterectomy specimens were fixed in formalin for 24–48 h; 3–4 mm-thick sections of the tumor were obtained on gross examination, including the area of deepest myometrial invasion. The specimens were dehydrated and embedded in paraffin. Four-µm-thick sections were obtained from the paraffin-embedded tissue blocks by microtome, mounted on glass slides, and stained with hematoxylin and eosin. All histological slides were reviewed by three pathologists with expertise in gynecological pathology (GFZ, DA and AT) to confirm the presence of MELF pattern. The MELF pattern of invasion was defined as the presence of microcystic, elongated, and/or fragmented glands with eosinophilic cytoplasm accompanied by acute inflammation, with or without a surrounding myxoid stroma [26]. Other histopathological parameters assessed were LVSI, categorized as “absent” (no LVSI), “focal” (a single focus was recognized) and “substantial” (>1 focus) [33]; a depth of myometrial invasion was categorized as less or more than 50% of the myometrial thickness. Sentinel lymph nodes were analyzed by using the ultrastaging technique, consisting of multiple serial sections cut at 150-µm intervals, examined with hematoxylin and eosin stain and cytokeratin (CK) AE1/AE3-immunostained sections, until all the lymph node was exhausted [34]. Lymph node involvement was categorized as isolated tumor cells (ITC, <0.2 mm), micrometastasis (between 0.2 and 2 mm) or macrometastasis (≥2 mm). Immunohistochemistry was performed with anti-L1CAM antibody (mouse monoclonal antibody, clone UMAB48, Leica Biosystems; dilution 1:200) by using a Leica Bond Max III platform (Leica Biosystems, Nussloch, Germany). L1CAM expression was categorized as: “absent” (i.e., no positive cells), “occasional cells” (positivity in <1% of cells), “low” (positivity in 1–9% of cells), “moderate” (positivity in 10–49% of cells), or “high” (positivity in ≥50% of cells). Moderate to high expression was labeled “overexpression”. Immunohistochemistry for p53 and mismatch repair proteins was performed as previously described [35]. In brief, antibodies against p53 (clone Do-7; ready to use; Leica), MLH1 (clone ESO5; ready to use; Leica), MSH2 (clone 79H11; ready to use; Leica), MSH6 (clone EP49; ready to use; Leica), and PMS2 (clone EPS1; ready to use; Leica) were used by using the Leica Bond III automatized platform (Leica Byosystems, Wetzlar, Germany), by following the manufacturer’s instructions. The expression of p53 was categorized as “wild-type” or “abnormal” according to the criteria proposed by Kobel et al. [36]. MMR expression was categorized as “proficient” or “deficient” according to the criteria proposed by the British Association of Gynaecological Pathologists [37]. Immunohistochemical analyses were performed by three pathologists with expertise in gynecological pathology (GFZ, DA and AT).

Univariate and multivariate logistic regression analyses were used to assess the association of pathological variables (depth of myometrial invasion, LVSI, p53, and MMR status) with lymph node involvement. A *p* value < 0.05 was considered statistically significant. Negative and positive predictive value were calculated. Statistical analysis was performed by using IBM SPSS Statistics, Version 26.0.

## 3. Results

**Figure 1:**
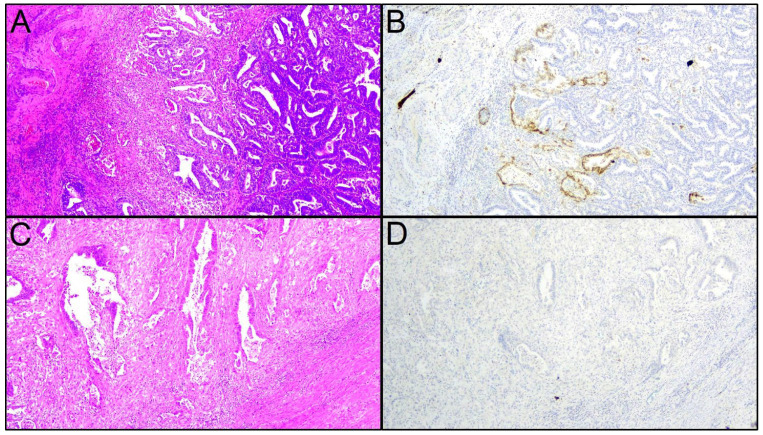
Microcystic, elongated and fragmented (MELF) pattern of invasion in endometrial carcinoma. (**A**) Low-grade endometrioid carcinoma with a MELF pattern on the invasion front on the left (hematoxylin-eosin; magnification ×40). (**B**) L1CAM overexpression in the MELF component (L1CAM immunohistochemistry; magnification ×40); please note that L1CAM positivity makes MELF glands more evident compared to the routine hematoxylin-eosin stain of the panel (**A**). (**C**) Detail of MELF glands at the invasion front of a low-grade endometrioid carcinoma (hematoxylin-eosin; magnification ×200). (**D**) Detail of MELF glands lacking L1CAM expression (L1CAM immunohistochemistry; magnification ×200).

Fifty-eight cases of presumed uterine-confined, low-grade endometrioid carcinoma with a MELF pattern of invasion were included in our study. The mean patient age was 64.8 years (range 43–86 years). Forty-one cases (72.4%) showed deep myometrial invasion; 14 cases (24.1%) showed focal LVSI, and 34 cases (58.6%) showed substantial LVSI. P53 immunohistochemical expression was wild-type in all cases; 17 cases (29.3%) showed MMR deficiency. Positive lymph nodes were found in 20 cases (34.5%), including 6 cases of ITC (10.3%), 6 cases of micrometastasis (10.3%) and 8 cases of macrometastasis (13.8%) (Table 1). None of the cases had an overall expression of L1CAM in more than 10% of the whole tumor. However, L1CAM expression was detected in the MELF component in 38 cases (65.5%), ranging from positivity in occasional cells to diffuse positivity (Figure 1). An expression of L1CAM in ≥ 10% of the MELF component was found in 24 cases (41.4%) (Table 1) and was the only significant predictor of lymph node involvement on both univariate (*p* < 0.001) and multivariate analysis (*p* < 0.001) with a negative predictive value of 81.6% and a positive predictive value of 80%. Because the significance of ITCs is undefined, we repeated the analysis after considering ITC cases as negative. Again, we found that an expression of L1CAM in ≥10% of MELF glands was the only significant predictor of lymph node micro-/macrometastasis on both univariate (*p* = 0.001) and multivariate analysis (*p* = 0.004) (Table 2).

## 4. Discussion

In our study, we aimed to assess the relationship between L1CAM expression and the MELF pattern of invasion in endometrial carcinoma, as they are both related to epithelial to mesenchymal transition and aggressiveness. Interestingly, none of the included cases showed L1CAM positivity in more than 10% of the whole tumoral area. However, almost two thirds of the tumors showed L1CAM expression in the MELF glands, which ranged from weak and focal to strong and diffuse. This first result suggests that L1CAM may be involved in the epithelial to mesenchymal transition process which underlies the MELF pattern of invasion, potentially being useful as a marker of MELF glands. Then, we used the threshold of 10% to define L1CAM overexpression in MELF glands. We found that L1CAM overexpression in MELF glands was significantly associated with lymph node involvement. In particular, L1CAM was the strongest predictor of lymph node involvement and was independent from LVSI, deep myometrial invasion, and MMR status. This finding suggests that the tendency of the MELF glands to spread to lymph nodes may depend on the degree of L1CAM expression. In the previous study by Stelloo et al., L1CAM showed unfavorable prognostic value in a cohort of endometrioid carcinomas including low-grade tumors with deep myometrial invasion and high-grade tumors with superficial myometrial invasion. Although a 10% threshold was adopted, such a percentage was assessed on the whole tumoral area and did not consider where L1CAM was expressed [14]. Our results suggest that not only the overall percentage, but also the localization of L1CAM expression could be worth consideration. It might be hypothesized that the conflicting results about the prognostic value of the MELF pattern in the literature [18,28,29,31] reflect differences in L1CAM expression. 

Previous studies have shown that L1CAM is often associated with p53 abnormalities [38]. In our series, all tumors were low-grade endometrioid carcinomas, which typically lack *TP53* mutations [8]. Indeed, all cases were p53 wild-type on immunohistochemistry. In these cases, the expression of L1CAM might be modulated by other molecular pathways not associated with p53. For instance, it has been shown that the Wnt/β-catenin pathway (which is often altered in low-grade endometrioid carcinomas [7]) may regulate L1CAM expression [39].

Noticeably, L1CAM overexpression in MELF glands predicted lymph node involvement with a positive predictive value of 80% and a negative predictive value of 81.6%; such data support a possible role in directing patient management. The evaluation of L1CAM expression in MELF glands may be integrated in the prognostic stratification of endometrial carcinoma, regardless of the overall expression in the whole tumor. For instance, a L1CAM expression ≥10% in MELF glands might have a prognostic significance similar to LVSI; uterine-confined, low-grade endometrioid carcinoma with L1CAM positive glands might be included in the high-intermediate risk category according to the ESGO-ESTRO-ESP risk classifier [11]. Further studies are necessary to assess these aspects.

Remarkably, all cases in our series were low-grade endometrioid carcinoma with no direct extension beyond uterus. Such homogeneity in our sample is a strength of our study. Furthermore, all cases in our series fell into the NSMP or the MMR deficient group, which are considered groups at intermediate prognosis, as discussed above [5,8,10,11]. It might be postulated that the MMR deficient signature has a different prognostic significance from the NSMP signature, as suggested by data regarding high-grade endometrial carcinomas. In fact, in high-grade carcinomas, MMR-deficient cases seem to have a better prognosis than NSMP cases [8,14]. Evidence in this regard has been found for clear cell carcinoma [40], mixed carcinomas [41], and carcinosarcoma [42]. Moreover, it has been suggested that all MMR deficient endometrial carcinomas may have a similar prognosis regardless of the histotype [8]; the only exception in this regard is represented by undifferentiated/dedifferentiated carcinoma, in which the immunohistochemical expression of SWI/SNF complex proteins seems to be more prognostically relevant than the MMR status [19]. Although the difference in MMR status in our series might appear as a limitation to the results, our multivariate analysis showed that the predictive value of L1CAM expression for lymph node involvement was independent from MMR status. Similarly, our cases varied with regard to depth of myometrial invasion and LVSI; however, these factors were found not to affect the results on multivariate analysis.

## 5. Conclusions

In conclusion, L1CAM is often expressed in MELF glands of endometrioid carcinoma, suggesting its involvement in the development of the MELF pattern. L1CAM positivity in ≥10% of cells in the MELF component, regardless of the overall expression in the whole tumor, appears as the strongest independent predictor of lymph node involvement in MELF cases. This may suggest that the prognostic value of MELF pattern depends on L1CAM expression. On this account, quantifying L1CAM expression in MELF glands might be prognostically relevant and might potentially impact the patient management. Further studies are warranted in this regard.

## Figures and Tables

**Table 1:** Summary of clinical, histological and immunohistochemical characteristics.

**Sample Size**	*n* = 58
**Age**	mean 64.8 years (range 43–86 years)
**Myometrial Invasion** - <50% - ≥50%	16 (27.6%) 42 (72.4%)
**LVSI** - absent - focal - substantial	10 (17.2%) 14 (24.1%) 34 (58.6%)
**p53 Status** - wild-type - abnormal	58 (100%) 0 (0%)
**MMR Status** - proficient - deficient	41 (70.7%) 17 (29.3%)
**Lymph Node Status** - negative - isolated tumor cells - micrometastasis - macrometastasis	38 (65.5%) 6 (10.3%) 6 (10.3%) 8 (13.8%)
**L1CAM in MELF** - absent - occasional cells (<1%) - low (1–9%) - moderate (10–49%) - high (≥50%)	20 (34.5%) 4 (6.9%) 10 (17.2%) 10 (17.2%) 14 (24.1%)

**Table 2:** Summary of histopathological and immunohistochemical features with regard to lymph node status and results of univariate and multivariate logistic regression.

**Variable**	**Lymph Node Status**		***p*** **-Value**	
	**Negative (*n* = 38)**	**Positive (*n* = 20)**	**Univariate**	**Multivariate**
Myometrial invasion ≥50%	25/38 (65.8%)	17/20 (85%)	0.120	0.577
Substantial LVSI	14/38 (36.8%)	10/20 (50%)	0.646	0.464
L1CAM ≥10% of MELF component	7/38 (18.4%)	16/20 (80%)	<0.001	<0.001
MMR-deficiency	12/38 (31.6%)	5/20 (25%)	0.601	0.815
**Variable**	**Lymph Node Status**		***p*** **-Value**	
	**Negative/ITC (*n* = 44)**	**Micro-/Macrometastasis (*n* = 14)**	**Univariate**	**Multivariate**
Myometrial invasion ≥50%	30/44 (68.2%)	12/14 (85.7%)	0.201	0.690
Substantial LVSI	16/44 (36.4%)	8/14 (57.1%)	0.934	0.978
L1CAM ≥10% of MELF component	12/44 (27.3%)	11/14 (78.6%)	0.001	0.004
MMR-deficiency	14/44 (31.8%)	3/14 (21.4%)	0.457	0.849

## Data Availability

Additional data are available from the corresponding author upon reasonable request.
